# Differential Refractometric Biosensor for Reliable Human IgG Detection: Proof of Concept

**DOI:** 10.3390/bios12070515

**Published:** 2022-07-12

**Authors:** João P. Mendes, Luís C. C. Coelho, Pedro A. S. Jorge, Carlos M. Pereira

**Affiliations:** 1 Centro de Investigação em Química UP (CIQUP)—Instituto de Ciências Moleculares (IMS), Departamento de Química e Bioquímica, Faculdade de Ciências da Universidade do Porto, Rua do Campo Alegre, 687, 4169-007 Porto, Portugal; joao.p.mendes@inesctec.pt (J.P.M.); cmpereir@fc.up.pt (C.M.P.); 2 INESC TEC—Instituto de Engenharia de Sistemas e Computadores, Tecnologia e Ciência, Departamento de Física e Astronomia, Faculdade de Ciências da Universidade do Porto, Rua do Campo Alegre, 687, 4169-007 Porto, Portugal; pedro.jorge@fc.up.pt; 3 Departamento de Química e Bioquímica, Faculdade de Ciências da Universidade do Porto, Rua do Campo Alegre, 687, 4169-007 Porto, Portugal; 4 Departamento de Física e Astronomia, Faculdade de Ciências da Universidade do Porto, Rua do Campo Alegre, 687, 4169-007 Porto, Portugal

**Keywords:** refractometric platform, optical sensing, long period fiber gratings, molecular imprinting, IgG antibodies

## Abstract

A new sensing platform based on long-period fiber gratings (LPFGs) for direct, fast, and selective detection of human immunoglobulin G (IgG; Mw = 150 KDa) was developed and characterized. The transducer’s high selectivity is based on the specific interaction of a molecularly imprinted polymer (MIPs) design for IgG detection. The sensing scheme is based on differential refractometric measurements, including a correction system based on a non-imprinted polymer (NIP)-coated LPFG, allowing reliable and more sensitive measurements, improving the rejection of false positives in around 30%. The molecular imprinted binding sites were performed on the surface of a LPFG with a sensitivity of about 130 nm/RIU and a FOM of 16 RIU^−1^. The low-cost and easy to build device was tested in a working range from 1 to 100 nmol/L, revealing a limit of detection (LOD) and a sensitivity of 0.25 nmol/L (0.037 µg/mL) and 0.057 nm.L/nmol, respectively. The sensor also successfully differentiates the target analyte from the other abundant elements that are present in the human blood plasma.

## 1. Introduction

Optical sensors are a very interesting analytical platform for fast and real-time detection [1,2]. In the last years, they have been applied in different fields such as medicine [3], general industrial [4,5], and environmental monitoring [6]. Taking different sizes and forms, optical sensors can assume a preeminent role to transform chemical or physical interactions between a sensing platform and an analyte into qualitative and/or quantitative information. Advanced methods targeting chemical and biological contaminants in water [7] or pathogens in health-care [8], are important to control the water quality or trace rapid and reliable medical diagnosis.

In this context, silica single mode optical fibers are robust, low-cost, corrosion resistant, have low attenuation at long distances, present immunity to electromagnetic interference, are easy to install and easy to move, allowing in situ measurements [9]. Its biocompatibility makes them suitable for chemical and biochemical functionalization, creating very interesting sensing schemes for the detection of specific targets such as viruses, drugs, proteins, antibodies, among others [10]. Additionally, some works using biofunctionalized optical fibers with specific receptors such as aptamers [11,12], antibodies or anti-antibodies [13,14] have been recently reported. However, if the production of the firsts presents drawbacks related with lack of simplicity and cost-effectiveness [15], the production of the others, which relies on the use of animals and/or cell culture, are time consuming, expensive and have limited shelf lifetime [16]. In this perspective, the efforts and progress achieved in the research of molecular imprinting techniques helped in the development of synthetic materials for specific recognition. Currently, molecularly imprinted polymers (MIPs) are widely used to mimic natural receptors, assuming different formats, sizes and thicknesses, ranging from nano- to micro-structures [17,18,19].

The most common technique to synthesize MIPs is the “Bulk Polymerization”. Briefly, the imprinted material is polymerized around the template molecule (target analyte) which is posteriorly removed. The resulted bulk polymers are crushed mechanically to a suitable size, resulting in small particles with specific recognition sites [20]. However, this technique has some disadvantages due to the crushing process, leading to irregular shapes, heterogeneous particle size, and partial or total destruction of the sites [21]. Despite those drawbacks, some researchers have been reporting fluorescent sensors based on MIPs produced by this method, by doping the resulted beads with amine-reactive groups according with the target analyte [22,23]. Besides, have been reported some progresses to overcome the problem of the quality and reproducibility in the production of these synthetic materials, by improving the polymerization techniques. *Pluhar* et al. used a two-phase technique, applying a dispersed-phase to polymerize spherical particles and a mini-emulsion phase to control the particle size avoiding agglomerations, producing pepsin selective MIPs. The performance of the resulted sensing structures was evaluated by UV-Vis spectrometry [24].

Moreover, MIPs produced on a nanoscale are also of particular interest, displaying a closer similarity to the natural antibodies: reduced number of binding sites per particle, fast kinetics of interaction, and high affinities and selectivity [25,26]. The solid-phase synthesis has been applied to produce artificial receptors for various diagnostic and life science applications [27]. The process starts with the covalent immobilization of the template onto a suitable solid support (e.g., spherical glass beads). The modified solid support is placed into the monomeric solution and the polymerization initiates under specific conditions, resulting in the formation of uniform spherical nanoparticles around the templates. As main advantages, this technique allows the formation of just one binding site per particle (mimicking monoclonal antibodies) and the process to extract the template molecule allows the isolation of the high-affinity from the low-affinity nanoMIPs [28]. *Canffarota* et al. developed an ELISA-like format device for vancomycin detection, based on molecularly imprinted nanoparticles using solid-phase polymerization [29]. Other works involving pseudo-ELISAs using MIPs in substitution of natural antibodies can be found in literature [30,31,32].

Other interesting technique relies on surface imprinting via grafting polymerization. This method allows to create polymeric layers on planar or spherical (and cylindrical) substrates by direct polymerization (covalently or non-covalently) on the surface of the substrate (e.g., spherical particles, planar surfaces, optical fibers) in the presence of the template molecule (with or without immobilization) [33]. The grafting process comprises some advantages such as improved affinity interactions due to the fast mass transfer induced by high analyte mobility, better control of the shape and morphology [20]. *Riskin* et al. presented an enhanced sensitivity surface plasmon resonance (SPR) technology by grafting gold nanoparticles to detect different chemical compounds, *He* and his co-workers presented a MIP-coated optical fiber to evaluate dabrafenib, and *Cennamo* et al. applied a similar approach to develop a system for SARS-CoV-2 detection [34,35,36]. Overall, MIPs can be considered to be more versatile, cost-effective, and have extended shelf lifetime compared with the common antibodies. However, the lack of toxicity studies and their scarce use for practical applications leads to a demand for further investigations. Nevertheless, these synthetic molecules have been showing many potential advantages to developing a new family of biosensors, especially in optical sensing [37].

Considering this, researchers in the field of optical sensing have directed their works to combine MIPs together with optical transduction systems to develop new sensing platforms such as optical fibers-based biosensors [38,39,40]. Additionally, fiber-optic evanescent wave (FOEW) sensors, where the long-period fiber gratings (LPFGs) can be included, are an interesting and promising technology to develop MIP-based optical sensors for bio-applications [41,42]. However, despite the advantages linked to LPFGs for label-free detection, these optical structures can work as true multi-parameter devices, being sensitive to strain, torsion, and temperature changes [43,44]. Considering this, the optical response of the LPFGs-based sensors needs to be carefully evaluated, namely in biosensing. In this work, a new sensing platform is proposed to detect and evaluate the human immunoglobulin G antibody (IgG).

The conventional methods for IgG identification are based on antigen-antibody specificity using methods such as enzyme-linked immunosorbent assays (ELISA) or cell/tissue immunofluorescence [45]. Despite their high specificity and selectivity, those methods involve laborious procedures, have limited multiplexing options and, require centralized laboratory equipment and specialized personnel, besides the respective drawbacks associated with natural antibody production [46].

Several researchers, using different methods, have been reporting theirs works to the scientific community in other to improve IgG quantitative and/or qualitative evaluation. Yang et al. demonstrated a SPR gold-coated prism coupler sensor doped with a single layer of graphene to detect IgG molecules by antigen-antibody specificity in a lower range (0–250 ng/mL; unknown LOD); Shen et al. reported an electrochemical sensor based on functionalized carbon nanotubes with ionic liquid, detecting IgG molecules in a range of 0.1–15 ng/L (LOD, 0.02 ng/L); and Choi et al. showed a nanoporous hydrogel photonic crystal modified with protein A, displaying a range of operation from 0.5 to 10 mg/L (LOD, unknown) [47,48,49]. Despite some promising results, there are still visible lacks on information of cross-reactivity tests, and limit of detections. Further, probably MIPs’ greatest advantages are the expected enhancement of the reversibility and stability of the sensing platforms when compared to those using natural antibodies.

The sensing scheme proposed in this work combines long-period fiber gratings and molecularly imprinted polymers, produced by surface imprinting, carrying specific binding sites targeting the analyte of interest. The platform includes a real-time correction system, based on the non-imprinted polymer (NIP), allowing reliable detection of the target analyte. Although it is possible to find works using optical fibers for IgG detection, from the best of our knowledge there are no published works conjugating LPFGs and molecular imprinting for the detection of this specific target and involving a correction system using the non-imprinted polymer at the same time. The behavior of the sensor system was successfully evaluated, assessing its sensitivity and selectivity.

## 2. Materials and Methods

### 2.1. Chemicals and Instrumentation

Sulfuric acid (p.a. 95–97%; Merck) and hydrogen peroxide solution (30 wt. % in H_2_O; Sigma-Aldrich, St. Louis, MO, USA) were used to freshly prepared piranha solution in 3:1 ratio, respectively. Allyltrimethoxysilane (ATMS, 97%, RI@20 °C = 1.4036; Gelest) was used for SAM formation. Acrylamide (Aam, >99%; Sigma-Aldrich), N-terc-Butylacrylamide (TBAam, 97%; Sigma-Aldrich), 2-Hydroxyethyl methacrylate (HEMA, 97%; Sigma-Aldrich), N,N′-Methylenebis(acrylamide) (BISAam, 99%; Sigma-Aldrich), N-(3-Aminopropyl) methacrylamide hydrochloride (APMA, 96%; Sigma-Aldrich), ammonium persulfate (APS, ≥98%; Sigma Aldrich) and N,N,N′,N′-tetramethylethylenediamine (TEMED, 99%; Sigma-Aldrich) were used for the polymerization process. IgG from human serum (≥95%; Sigma-Aldrich) was used as a template molecule and as target to build and assess the sensor. Albumin from human serum (HSA; ≥97%, Sigma-Aldrich) and transferrin from human blood plasma (HTR; ≥95%, Sigma-Aldrich) were used as competitors. Phosphate buffered saline solution (PBS, 0.01 mol/L, pH 7.4; Sigma-Aldrich), deionized water (DIW; Wasserlab, type II Analytical Grade), ethanol (96%; LabChem), sodium dodecyl sulfate (SDS; Sigma-Aldrich), sodium chloride (NaCl; Sigma-Aldrich), and ethylene glycol (EG; >99%, Sigma-Aldrich) were also used. A standard single-mode optical fiber (SMF28, Corning^®^) and a BraggMetter unit (HBK, Fibersensing) were used to develop the sensor and to process the light signal, respectively.

### 2.2. Long-Period Fiber Grating

**Figure 1:**
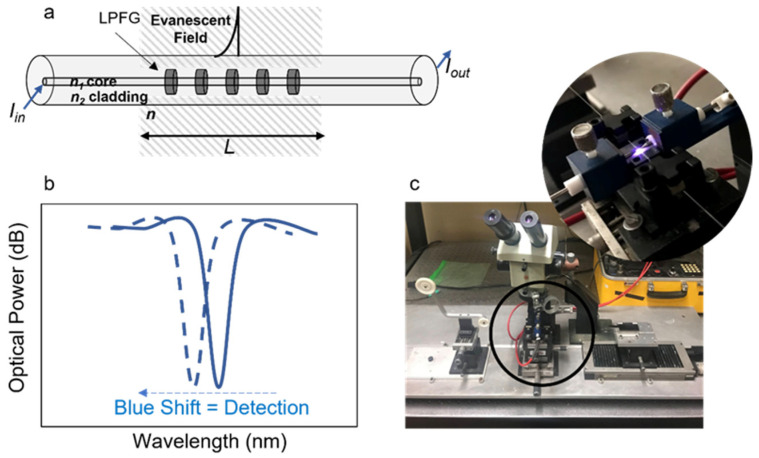
Schematic presentation of: (**a**) optical fiber with a long-period grating; (**b**) LPFG typical optical response (solid blue line) and blue shift (dashed blue line) due to refractive index change; and (**c**) picture of the LPFG microfabrication by electric arc setup, displaying the electric charge between two tungsten electrodes.

A set of LPFGs were fabricated by the induced arc-electric technique, as reported by Rego (2016) [50], applying point-by-point electric arc discharges using a current of 9 mA during 1 s, along 30 to 50 mm and a period of 415 µm, producing a modulation in the core refractive index (RI). The created optical structure works as a wavelength filter, presenting a spectrum with several attenuation bands which depends on the coupling conditions from the core propagating mode to the co-propagating cladding modes. Each of these attenuation bands corresponds to a different guided cladding mode and its wavelength position strongly depends on the external refractive index. In general, as the external RI (*n*) increases, the sensitivity of the LPFG enhances monotonically to a maximum value that is established by the RI of the cladding mode (*n*_2_). When *n* = *n*_2_ the cladding mode becomes unguided. If the RI of the core mode (*n*_1_) is not changed by *n*, the effect of the external RI around the cladding can be expressed by:

(1) $${\left(\frac{d\lambda }{d_{n}}\right)}_{m}=\left(\frac{d\lambda }{dn_{2,m}^{eff}}\right)\times \left(\frac{dn_{2,m}^{eff}}{dn}\right)$$

where λ and $n_{2,m}^{eff}$ are the transmitted spectra and the effective refractive index of the radial cladding mode (Figure 1), respectively [51]. Further information about the spectral characteristics of the LPFGs is presented in the Supplementary Material file.

### 2.3. Experimental Setup

**Figure 2:**
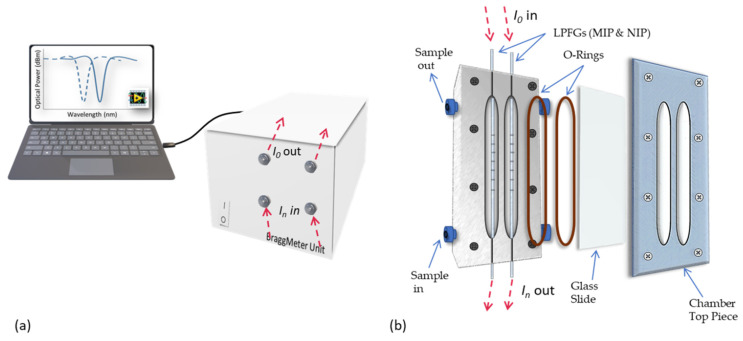
Experimental setup used for LPFG surface preparation, template extraction and sensor performance evaluation, including the interrogation unit (**a**) and the fluidic chamber components (**b**).

The LPFGs were placed under tension into a fluidic chamber (Figure 2) specially designed for this purpose. The fibers were connected to a BraggMeter unit working in transmission mode and the light signal was transformed in analytical data through a dedicated LabView routine in a spectral range from 1500 nm to 1600 nm. Furthermore, a second chamber includes a bare LPFG to explore its behavior under the same experimental conditions.

### 2.4. Step-by-Step of Sensor (bio)Chemical Preparation

#### 2.4.1. Fiber Surface Modification

Silanization processes are commonly used to chemically modify silica optical fibers [52,53]. In this work, both LPFGs were cleaned by immersing the silica sensing region in the piranha solution for 30 min at room temperature. After washing several times with DIW, the optical fibers were placed in the oven for 2 h at 60 °C to remove all the water from its surface and generate hydroxyl groups (-OH). This step is extremely important to the success of the following reaction between the silicon group of the ATMS with the -OH previously created, in order to cover the fiber surface as much as possible. The LPFGs were placed into the fluidic chamber where the sensing region was isolated from the external medium. The chamber was filled with a 2% ATMS solution in an ethanol/water mixture (96% ethanol) and reacted for 16 h at 4 °C, allowing a SAM formation in all the fiber sensing region. The sensing surface was washed with fresh ethanol and DIW for several times and dried for 2 h at 60 °C enabling covalent bonds formation (see Supplementary Material, Figure S1). This chemical modification will help to hold the polymeric layer attached to the LPFG surface by C-C bonds. In order to observe the progress of the fiber surface modification, the spectra after each introduced change on the LPFG were acquired (in DIW): H_2_O (reference); H_2_O@Allylsilane (after silanization process); H_2_O@Polymerization (after MIP layer formation); and H_2_O@TemplateExtracted (after template extraction).

#### 2.4.2. MIP/NIP Layer Formation and Template Extraction

**Figure 3:**
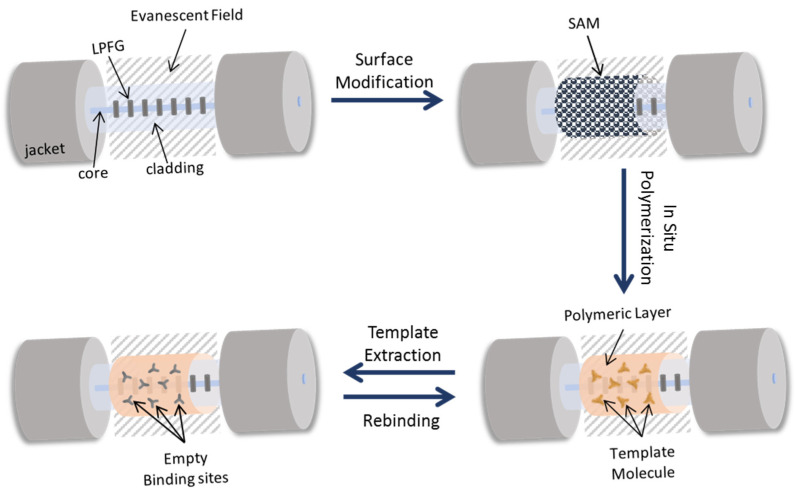
Schematic figure of the in-situ polymerization, sequentially showing the process of the fiber surface chemically modification from the allylation to the template extraction. NIP-coated LPFG has the same procedure without the presence of the template and, consequently, template extraction step.

The synthesis of the molecularly imprinted polymer layer was adapted from a recently reported strategy for molecular imprinting [36]. A pre-polymeric mixture including Aam (0.68% *w*/*v*), TBAm (0.24% *w*/*v*), HEMA (0.1% *v*/*v*), BIS (0.58 *w*/*v*), and APMA (0.12% *w*/*v*) in 10 mmol/L PBS solution, was prepared. After mixing all components, the pre-polymeric mixture was ultrasonicated for 15 min and bubbled with nitrogen (N_2_) for 1 h. After that, the template was added to the solution in a final concentration of 5 × 10^−7^ M followed by the addition of APS (0.2% *w*/*v*). Then, 1 mL of the mixed solution was dropped onto the LPFG followed, immediately, by a suitable amount of TEMED over the sensing section, performing a final concentration of a 0.12% (*v*/*v*), to catalyze the reaction. The reaction started immediately, and the polymerization took place for, approximately, 10 min being stopped by washing the LPFG surface with copious amounts of DIW. For the template extraction, the sensing region was immersed in DIW at 60 °C for 1 h (replacing the warm water at each 5 min) and then washed with a 5% SDS solution (in PBS) for 10 times. Finally, the LPFG was immersed in a 0.5 M NaCl solution (in 5% SDS) overnight, washed three times with SDS and five times with DIW. After template extraction, the MIP layer built on the LPFG surface was characterized and sensitivity and selectivity towards human IgG antibodies was evaluated. Figure 3 exemplifies the polymerization process. The interaction receptor/target analyte was evaluated on an LPFG covered with a non-imprinted polymer layer (NIP). The NIP layer was built in the same conditions of the MIP without adding the template.

### 2.5. Binding Experiments and Selectivity Evaluation

For the binding experiments, standard solutions of IgG antibody (Mw = 150 KDa, [54]) in PBS (pH 7.4) were prepared at different concentrations, ranging from 1 to 100 nmol/L. The LPFGs, MIP-coated and NIP-coated, were immersed in buffer solution and the respective spectra were acquired (reference spectra). Each IgG concentration was placed over the sensing region for 5 min followed by the washing process with buffer solution (5×). Then, the respective spectra were acquired also in buffer solution to evaluate the wavelength shift (Δλ) considering the initial position measured in the buffer solution. The spectra of the NIP-coated LPFG were used to correct RI changes caused by “bulk effect” and/or temperature and/or tension changes, subtracting its Δλ to the Δλ of the MIP-coated LPFG. The non-imprinted layer Δλ was also tested in the same conditions versus the Δλ of a bare LPFG fiber to understand the impact of the nonspecific RI variations. The selectivity (MIP vs. NIP vs. (MIP-NIP)) of the optical platform was also accessed using a single concentration for the competitors in presence of half that concentration for the IgG antibody.

## 3. Results & Discussion

### 3.1. LPFG Sensitivity at Refractive Index Variations

The LPFG sensitivity was assessed by measuring different refractive index solutions attained by mixing DIW and EG in different portions. The refractive index at 589.3 nm (sodium D line) of each solution was evaluated by a digital refractometer (DR-A1, Atago CO., LTD, Bellevue, WA, USA) displaying, increasing the EG concentration, the refractive indices from 1.3322 (DIW) to 1.3751. Each solution was placed over the sensing region for 5 min before spectra acquisition. The mean of 10 spectra acquired for each solution (after exposure time) as well as the calibration curve of the experimental values obtained by fitting the respective spectra with a Gaussian curve versus the wavelength position are shown in the supplementary information (Supplementary Material, Figure S2). The sensitivity (S) of the LPFG is provided by the slope of the calibration curve that was about |S| = 130 nm/RIU. The Figure of Merit (FOM) is also a comprehensive parameter to evaluate the sensor performance. Thus, the FOM of the LPFG-based sensor was also assessed, displaying a value around 16 RIU^−1^ in the RI range from 1.3322 to 1.3658 (see Supplementary Material; Equation (S1) and Figure S2.

### 3.2. Step-by-Step of Sensor Preparation

**Figure 4:**
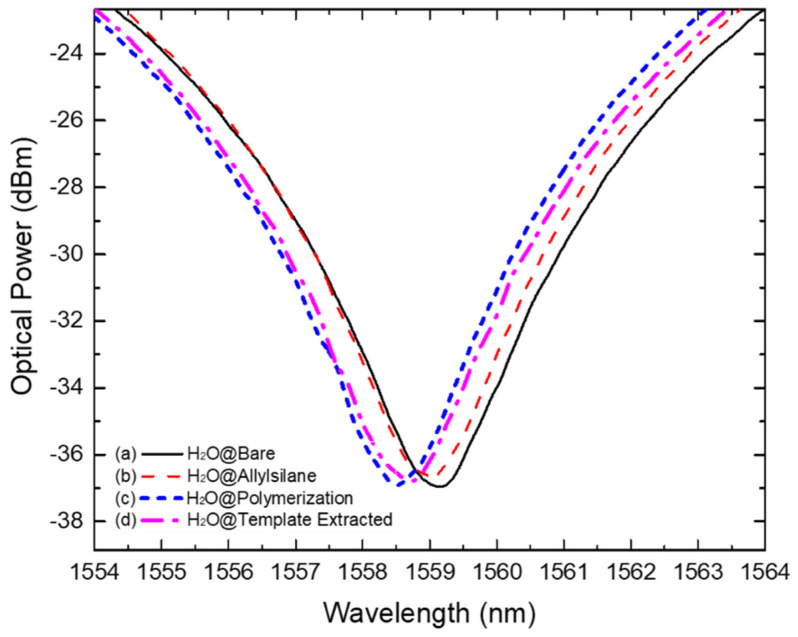
Acquired spectra showing the wavelength resonance band position resulted from measurements in pure water of (a) bare LPFG (solid black line); (b) allyl-silanized LPFG (dashed red line); (c) MIP-coated LPFG (short dashed blue line); and (d) MIP-coated LPFG after template extraction.

In the allylation process, a self-assembled monolayer (SAM) was covalently formed at the LPFG surface causing a variation of the RI (by increasing it) in the surrounding medium of the optical fiber. This RI increment is proved by the resonance wavelength blue shift (125 pm to lowest wavelengths) observed in Figure 4b which confirms the layer formation. The same behavior was observed by *Gupta* after (3-aminopropyl)triethoxysilane (APTES) immobilization on a LPFG surface for posterior soil fungi detection [55].

**Figure 5:**
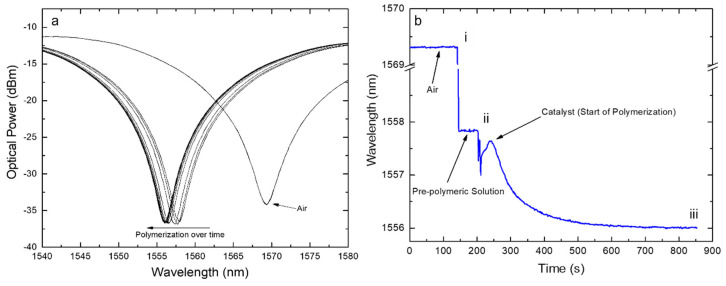
(**a**) Position of the spectrum changing over time and (**b**) tracking of the band position versus the deposition time where: (i) is the time when the polymeric solution was dropped onto the LPFG followed by the consequent shift due to RI change in the surrounding medium; (ii) is the time when the catalyst was placed on the previous solution followed by the beginning of the polymerization process; (iii) is the time when the polymerization process was interrupted (after about 10 min from step (ii).

Moreover, a shift was also observed resulting from the polymerization process. The spectral evolution of the polymeric layer formation was controlled in real-time by monitoring the LPFG wavelength shift during all the process (Figure 5). As a result of the polymerization and further washing process, the resonance wavelength band stabilized, fixing its position at 575 pm and 450 pm away from the H_2_O and the H_2_O@Allylsilane spectra, respectively (Figure 4c). This result match the observation reported by *Arcadio* et al. for a surface plasmon resonance (SPR) sensor for bovine serum albumin (BSA) detection [56]. Similarly, *Verma* and *Gupta* used a similar approach to develop a MIP layer onto a silver-coated silica optical fiber for antibiotic recognition using the SPR phenomenon [57].

After template extraction, as described in Section 2.3, a decreasing of the RI around the LPFG (shifting 300 pm to higher wavelengths) was observed which can be related to the IgG molecules removal from the MIP layer (Figure 4d). In literature, similar procedures to remove electrostatically immobilized templates using NaCl were reported by other authors, e.g., by Schwark et al. to remove IgG molecules from imprinted macroporous membranes; Matsumoto et al. to remove prostate-specific antigen (PSA) from a synthesized MIP layer on an SPR chip; and by Yang et al. when developing a selective and sensitive impedance sensor targeting the BSA protein, using a NaCl/SDS solution in the template extraction stage [58,59,60]. The resulted MIP receptors were tested in the presence of IgG antibodies and competitors. The NIP formation process was carried out in the same experimental conditions and the resulted data is exhibited in Figure S3 (Supplementary Material).

### 3.3. Binding Experiments

**Figure 6:**
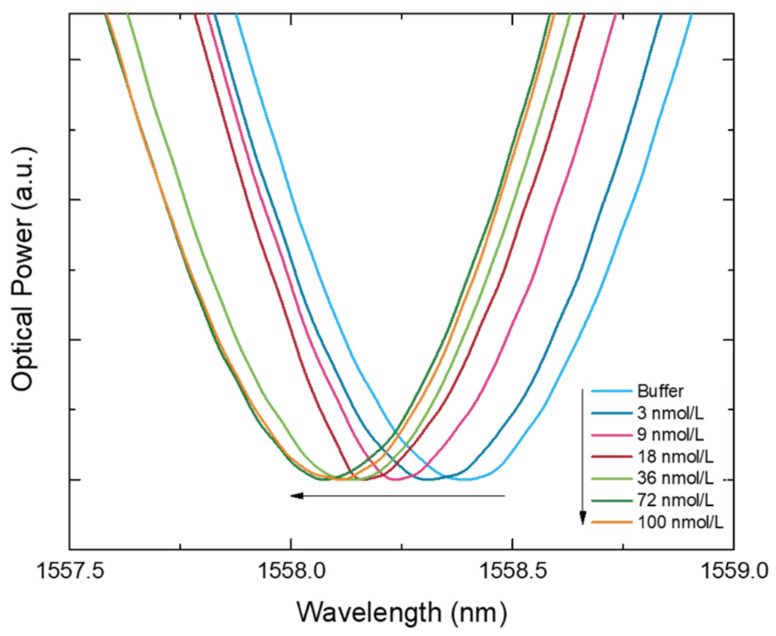
Transmitted spectra (normalized to the minimum) shifting according with the increase of IgG concentration (*n* = 10). Some curves were removed (1; 6; and 13 nmol/L) in order to provide the clearest image for a better evaluation of the spectral behavior.

The binding experiments were carried out placing the MIP-coated and the NIP-coated LPFGs in different grooves but using the same standard IgG solution to assess its spectral behavior. The exact position of the wavelength band is given by a gaussian fit. The bare LPFG was also exposed to the same standard solutions and its Δλ was also evaluated. In that way, Figure 6 displays the MIP-coated LPFG transmitted spectra for different concentrations of IgG antibodies and reveals a systematic blue shift of the resonance wavelength when the analyte concentration increases.

**Figure 7:**
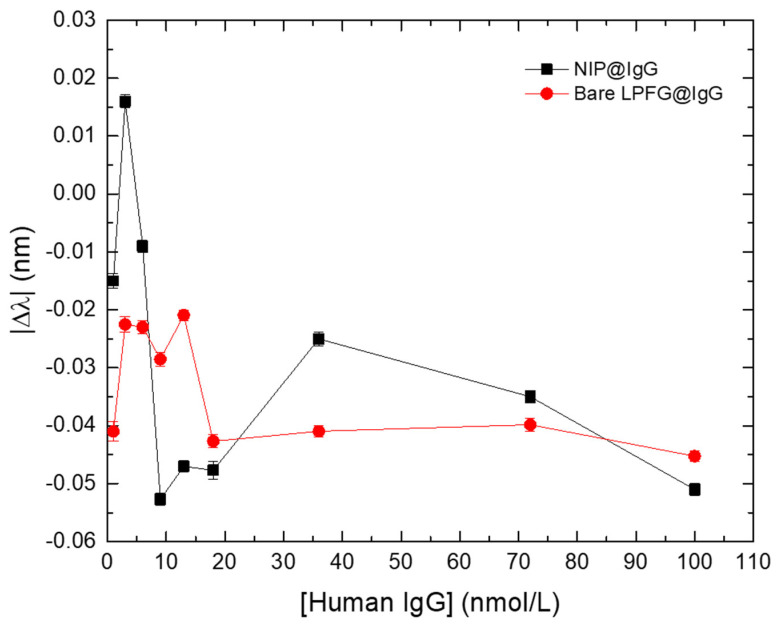
Wavelength variation (Δλ) of the NIP-coated LPFG (black squares) and the bare LPFG (red circles) in the presence of different IgG concentrations. Errors bars were obtained from standard deviations.

To understand the impact, on the LPFGs, of the RI deviations of the external medium, caused by “bulk effect” and/or temperature fluctuations, the responses (in the format of Δλ) of the NIP-coated LPFG (NIP@IgG) and the bare LPFG (Bare LPFG@IgG) were measured and compared (Figure 7).

Figure 7 displays the results of Δλ (after correcting for the offset values, according to respective LPFGs sensitivities) for the NIP-coated LPFG and the bare LPFG. Evaluating the results, obtained in the presence of the same analyte solutions, it is possible to conclude that they display similar behaviors. However, it is also noticeable that, along with the similar general trend, there are fluctuations on Δλ that could be attributed to external effects (e.g., temperature variations, physical tension) as well as to “bulk effect” for the NIP-coated LPFG.

**Figure 8:**
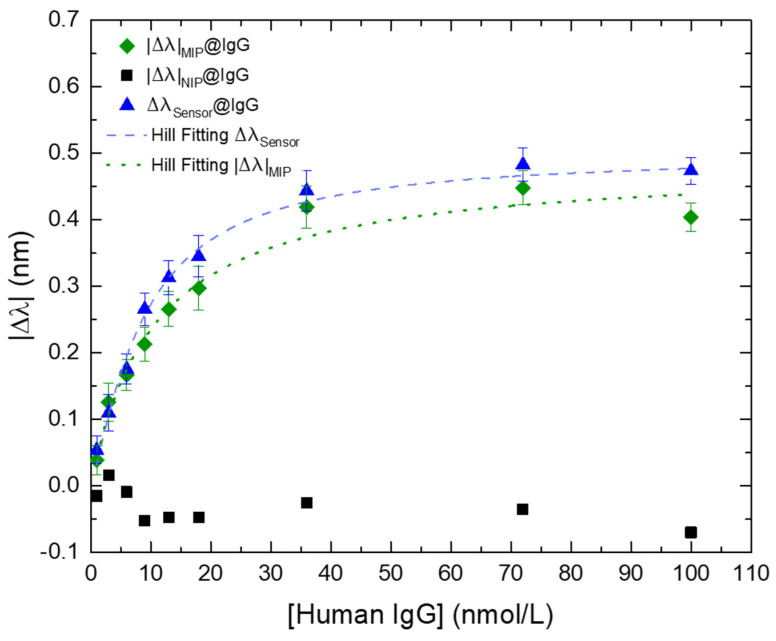
Wavelength variation (Δλ) of the MIP-coated LPFG (green diamonds), NIP-coated LPFG (black squares), and the differential “MIP-NIP” (blue triangles) in the presence of different IgG concentrations. The dashed blue line and the pointed green line are the respective fittings, using the Hill model equation (Equation (S2), Supplementary Material), of the MIP-coated LPFG and NIP-coated LPFG. Errors bars were obtained from standard deviations.

Considering the results from Figure 7, this configuration allows a correction to external parameters in real time making the interaction of the MIP-coated LPFG with different concentrations of IgG antibodies (MIP@IgG) with increased reliability of the sensing scheme. The proposed correction consists in subtracting the response of the NIP-coated LPFG to the signal of the MIP-coated LPFG layer, according with:

(2) $$\Delta \lambda_{\mathrm{sensor}}={\left|\Delta \lambda \right|}_{\mathrm{MIP}}-{\left|\Delta \lambda \right|}_{\mathrm{NIP}}$$

where Δλ_sensor_ is the differential mode between the modules of the MIP-coated and NIP-coated LPFGs wavelength shifts, |Δλ|_MIP_. is the module of the wavelength shift of the MIP-coated LPFG, and |Δλ|_NIP_ is the module of the wavelength shift of the NIP-coated LPFG. Raw experimental data and corrected signal are plotted on Figure 8.

It is noticeable that the Δλ_Sensor_ mode reveals a better fitting to the Hill-Langmuir model, improving the performance of the sensor targeting the analyte and providing more reliable results and this is a clear demonstration of the importance of using the NIP-coated LPFG for real time correction of disturbances caused by “bulk effect” and external influences in the measured solution.

Table 1 shows the statistical data from both fittings, revealing a small Residual Sum of Squares (RSS) proving that the differential “MIP-NIP” is the better model to use in this kind of measurements (for residual plots see Supplementary Material, Figures S4 and S5).

**Figure 9:**
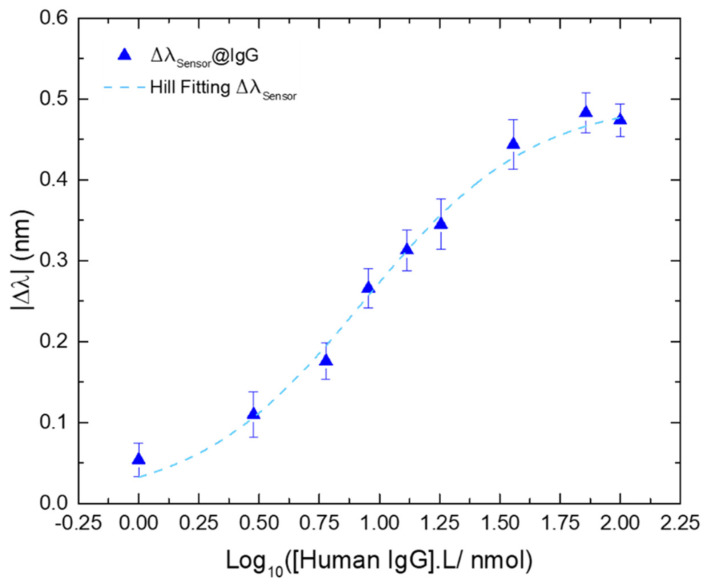
Wavelength variation of the differential Δλ_Sensor_@IgG, as a function of the logarithmic concentration of IgG (blue triangles); and the respective Hill fitting (R^2^ = 0.9912; dashed blue line). Errors bars were obtained from standard deviations.

However, to assess the full sensitivity of the sensing platform we need to resort to the Hill-Langmuir equation by plotting the experimental data in the lin-log form, i.e., the wavelength variation (in nm) versus the logarithm of the IgG concentration (in nmol/L), to achieve symmetrical confidence intervals for the experimental parameters [61,62,63]. Therefore, the respective Hill fitting (Figure 9) was plotted through Equation (3), where **∆λ*_c_*** is the wavelength variation at the concentration ***C***; **∆λ*_max_*** is the wavelength variation at the saturation point (or response to the infinite); ***K*** is the ligand concentration at the 1/2Δλ*_max_*; and ***n*** is the Hill coefficient, with: *K* = 8.61 L/nmol, *n* = 1.24, and a Δλ*_max_* = 0.490 nm.

(3) $$\Delta \lambda_{c}=\frac{\Delta \lambda_{max}}{1+10^{n\left(\log K-\log C\right)}}$$

The calculated sensitivity was |S| = 0.057 nm.L/nmol (|S| = |∆λ|*_max_*/K), in the range from 1 to 36 nmol/L. The affinity constant (K_aff_) was also assessed revealing a value of K_aff_ = 0.12 L/nmol (K_aff_ = 1/K). The limit of detection (LOD) was calculated based on a proposed method for non-linear sensors, using the signal-to-noise (S/N) approach [64]. This method was suggested for an ion-selective electrodes (ISEs) model through Equation (S3) (see Supplementary Material). Adapting the LOD definition for non-linear sensors to Equation (2), considering that the ***n*** parameter is, geometrically, the factor that characterizes the slope of the curve at the midpoint and *σ* is the standard deviation of the blank, the LOD for this kind of curves can be defined as [63]:

(4) $$\mathrm{LOD}=\log K\left(10^{\frac{3\sigma }{n}}-1\right)$$

Therefore, the calculated LOD for the sensing platform proposed in this work was 0.25 nmol/L.

Additionally, the reversibility of the sensor was also evaluated, by applying the same protocol used for template extraction. However, as is shown in Supplementary Material (Figure S6), the sensor wasn’t totally regenerated (just about 82%) and further research needs to be done to improve the regeneration step.

Table 2 shows a comparison between the presented sensor with others in the literature for IgG (or anti-IgG; human or others) using different configurations, working ranges and LODs based on different configurations using optical fibers.

Additionally, the sensor proposed in this work is an easy device to build, requiring fewer protocols for its development. Furthermore, despite using synthetic materials for specific recognition, the proposed sensing platform offers a similar LOD and a significant resolution (considering the lowest evaluable concentration) compared to the majority of the sensors listed in Table 2. Moreover, the synthetic receptors are an advantage compared with the natural receptors considering the production requirements. Finally, the included correction system improves the sensor performance by offering a more accurate response.

Despite the difficulties in finding LPFG-based sensors coupled with MIPs for IgG detection, other sensing platforms such as electrochemical-based sensors or SPR-based sensors using planar gold chips, applying molecular imprinting for its selective detection have been reported. Table 3 compares some of those researches based on MIPs for biorecognition of the IgG molecule.

The parameters displayed in Table 3 show that molecular imprinting materials applied to biosensing can compete directly with the sensors based on antigen-antibody specificity. Moreover, the sensing device proposed in this work has similar performances, although more investigations need to be conducted in order to improve specificity and sensitivity to achieve similar performances that are presented by electrochemical sensors.

### 3.4. Selectivity Tests

**Figure 10:**
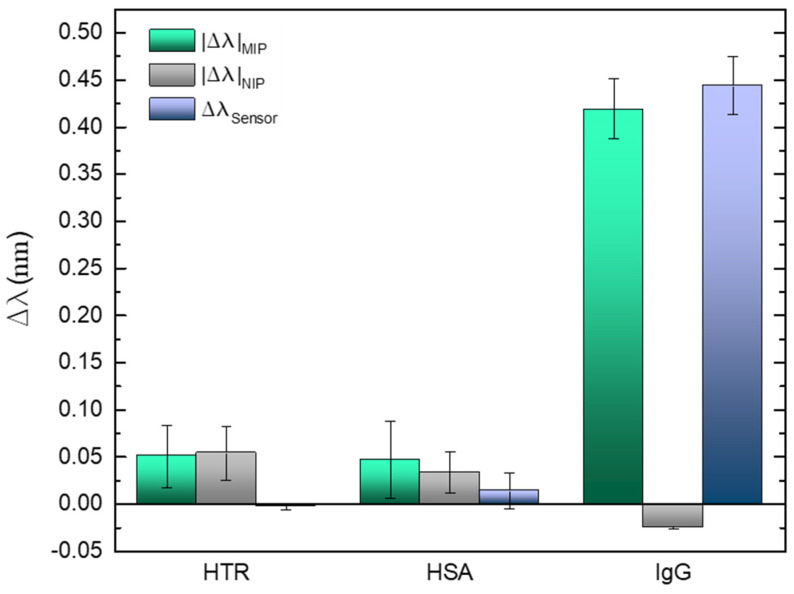
Selectivity evaluation in the in the presence of the competitors (HTR and HSA) and the target analyte (human IgG). Errors bars were obtained from standard deviations.

The selectivity of the sensing platform was tested in the presence of IgG competitors acting as interferents and selected due to their abundant presence in the human blood plasma as well as the IgG antibody. Therefore, the negative controls were performed with HSA protein [76] and the HTR glycoprotein [77]. Other authors such as Aylaz et al. or Ruiz et al. used the same blood plasma components to attest the selectivity of their platforms [78,79]. Figure 10 show the information resulted from the sensing region incubation in 60 nmol/L (in PBS) of each interferent and in 36 nmol/L of the IgG standard solution, for five minutes. Between each step, the LPFG was washed several times with the buffer solution. The results are presented as mean of 10 measurements acquired after the exposure time.

In case of the control tests, slight shifts are visible for each competitor comparing with the specific binding towards IgG. It is undeniable that the sensitivity and selectivity of the imprinted cavities in the presence of the IgG antibody, revealing a wide variation for about half of the concentration used for the interferents assays. The impact of the proposed correction system is also noticeable, reducing the response to the interferents in 30%, allowing reliable measurements by improving the rejection of “false positives”.

## 4. Conclusions

An optical (bio)chemical sensor system coupling long-period fiber gratings and molecular imprinted binding sites for specific detection of human IgG antibodies was developed and experimentally investigated. The sensor comprises the advantages associated to the optical fibers, combining them with the advantages linked to the molecular imprinting. The experimental results suggests that this platform reveals good performances in terms of selectivity. However, the sensitivity can be improved by reducing the polymerization time, increasing the possibilities to obtain a larger number of binding sites at the LPFG surface and avoid fast saturation, and by coating the LPFG surface by metal-oxide films to increase its sensitivity. It was noticed that the sensor recovery after template extraction is somewhat different from the shift observed after the calibration process. This situation could be explained by RI deviations or by the existence of low- and high-affinity binding sites. In the washing process, to remove non-reacted monomers, some IgG antibodies attached to low-affinity binding sites could be removed from the polymer surface. However, during calibration, those sites are still available for interaction with the analyte. Nevertheless, it is evident that the proposed label-free sensor, is based on a robust and emerging technique for MIP fabrication requiring a short incubation time for target detection. Moreover, the correction system seems to improve the sensor performance and its reliability, being a very attractive approach to be implemented in clinical diagnosis. Additionally, the sensing platform used in this work is simple to realize and employs green strategies for biological recognition. Furthermore, the implementation of the proposed system is not limited to being applied in the medical field. Its combination with low-cost interrogation units, with several reading channels, results in very attractive devices for multiplexing different analytes of interest in water quality control, namely water contaminants. Moreover, SPR-based optical fibers can be used to perform MIP/NIP differential measurements using a single optical fiber employing two distinct sensing regions (MIP-coated and NIP-coated). In this way, is possible to obtain miniaturized devices with high sensitivity and selectivity for reliable chemical and biological sensing.

## Figures and Tables

**Table 1:** Statistical data resulted from the respective Hill fittings of the curves presented in Figure 6.

Model	Reduced Chip-Sqr	Residual Sum of Squares	R-Square (COD)	Adj. R-Square
**|Δλ|_MIP_@IgG**	0.9189	6.4322	0.9762	0.9728
**Δλ_Sensor_@IgG**	0.4067	2.8470	0.9923	0.9912

**Table 2:** Comparative analysis of different platforms for human IgG detection based on optical fibers sensors.

Sensor Configuration	Transduction Method	Analyte (Receptor)	Detection Range	LOD	Ref.
TFBG modified with graphene oxide and staphylococcal protein A.	SPR	Human IgG (protein A)	30–100 µg/mL	0.5 µg/mL	[65]
Gold (Au) film coated photonic crystal fiber dopped with Au nanoparticles modified with protein A and anti-human IgG.	SPR + LSPR	Human IgG (anti-IgG)	1–30 µg/mL	0.037µg/mL	[66]
S-tapered fiber modified with dopamine and Protein A.	Mach-Zehnder interferometer	Human IgG (protein A)	0.25–2 µg/mL	0.028 µg/mL	[67]
Thin core single-mode fiber sandwiched two single-mode optical fibers modified by anti-IgG immobilization.	Mach-Zehnder interferometer	Human IgG (anti-IgG)	100–1000 µg/mL	not reported	[68]
Sol−gel-based titania−silica thin film coated LPFG modified by IgG immobilization.	LPFG	Anti-human IgG (human IgG)	0.001–100 µg/mL	0.013 µg/mL	[69]
Sol-gel-based titania-silica over coupled LPFGs modified by mouse IgG immobilization.	LPFG	Anti-mouse IgG (mouse IgG)	0.1–100 µg/mL	0.025 µg/mL	[70]
GO-coated-U-bent LPFG inscribed in a two-mode fiber modified by anti-human IgG immobilization.	U-bent LPFG	Human IgG (anti-IgG)	3–20 µg/mL	0.023 µg/mL	[71]
MIP-coated LPFG for Human IgG detection	LPFG	Human IgG (MIP layer)	0.15–15 µg/mL (1–100 nmol/L)	0.037 µg/mL (0.25 nmol/L)	this work

TFBG-tilted fiber Bragg grating; SPR-surface plasmon resonance; LPFG-long period fiber grating; GO-graphene oxide; LOD-limit of detection; Ref.-reference.

**Table 3:** Comparative analysis of different platforms for human IgG detection based on molecular imprinting.

Sensor Configuration	Transduction Method	Analyte (Receptor)	Detection Range	LOD	Ref.
SPR gold chip modified with a MIP nanofilm	SPR (planar gold chip)	F_ab_ fragment (MIP nanofilm)	2–15 µg/mL	0.056 µg/mL	[72]
nanoMIPs-coated SPR gold chip	SPR (planar gold chip)	IgG, F_c_ domain, peptide epitope (nanoMIPs)	0.003–1 µg/mL	not reported	[73]
Electrochemical biosensor based on graphene quantum dots covered with a MIP layer	Cyclic Voltammetry	IgG molecule (MIP layer)	10^−4^–0.05 µg/mL	2 × 10^−5^ µg/mL	[74]
MIP layer interfaced with a SAW chip	Surface Acoustic Wave	IgG molecule (MIP layer)	0.06–8 µg/mL	Not reported	[75]
MIP-coated LPFG for Human IgG detection	LPFG	Human IgG (MIP layer)	0.15–15 µg/mL (1–100 nmol/L)	0.037 µg/mL (0.25 nmol/L)	this work

## Data Availability

Not applicable.

## Supplementary Materials

The following supporting information can be downloaded at: https://www.mdpi.com/article/10.3390/bios12070515/s1, Figure S1: Schematic figure of the fiber surface allylation process; Figure S2: Spectral characterization of the optical fiber used for MIP/LPFG sensor; Figure S3: NIP polymerization timeline and spectra evolution; Figure S4: Residual Plots resulted from the Hill fitting of the MIP@IgG curve; Figure S5: Residual Plots resulted from the Hill fitting of the (MIP-NIP)@IgG curve; Figure S6: Wavelength peak position of the differential (MIP-NIP) shifting over time throughout reversibility trials. Equation S1: Figure of Merit (FOM); Equation S2: Hill model equation; Equation S3: Ion-selective electrodes (ISEs) model.
