# Supplementary material for: Differential Refractometric Biosensor for Reliable Human IgG Detection: Proof of Concept
Source: Biosensors (Basel). 2022 Jul 12;12(7):515. doi: 10.3390/bios12070515 (PMC9312733; doi:10.3390/bios12070515)
Supplement: Supplementary file 1 [file biosensors-12-00515-s001.zip › biosensors-1765144-supplementary.pdf]

Appendix A/Supplementary Material

Differential Refractometric Biosensor for Reliable Human IgG detection: Proof of Concept

João P. Mendes[1,2,3*], Luís C. C. Coelho[2,4*], Pedro A. S. Jorge[2,4] and Carlos M. Pereira[1,3]

[1]Chemistry Research Unit of University of Porto, Faculty of Sciences of University of Porto, 4169-007 Porto, Portugal; joao.p.mendes@inesctec.pt; cmpereir@fc.up.pt

2INESC TEC – Institute for Systems and Computer Engineering, Technology and Science, and Faculty of Sciences of University of Porto, 4169-007 Porto, Portugal; luis.c.coelho@inesctec.pt; pedro.jorge@fc.up.pt.

3Chemistry and Biochemistry Department, Faculty of Sciences of University of Porto, 4169-007 Porto, Portugal

[4]Department of Physics and Astronomy, Faculty of Sciences of University of Porto, 4169-007 Porto, Portugal

*Corresponding author: luis.c.coelho@inesctec.pt

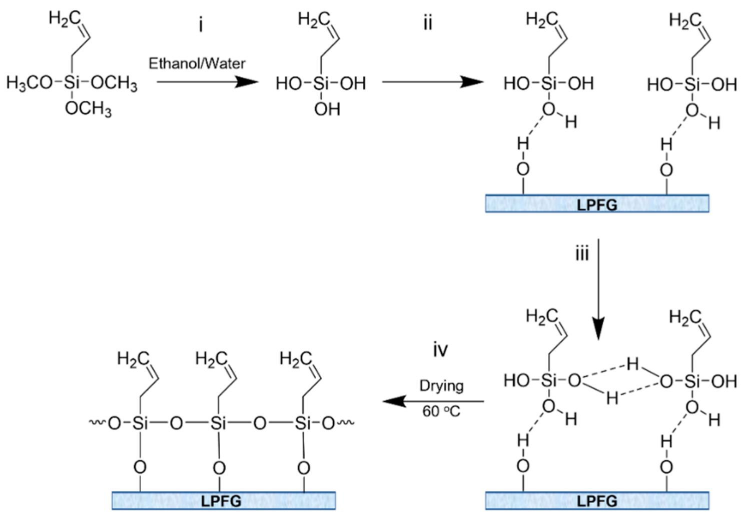

Supplementary figure S1 – Schematic figure of the fiber surface allylation process: i. hydrolysis (formation of -OH groups); ii. chemisorption (reaction of the allylsilane with the LPFG surface); iii. cross-polymerization (reaction between different allylsilane molecules at the LPFG surface); iv. covalent bond formation (monolayer formation).

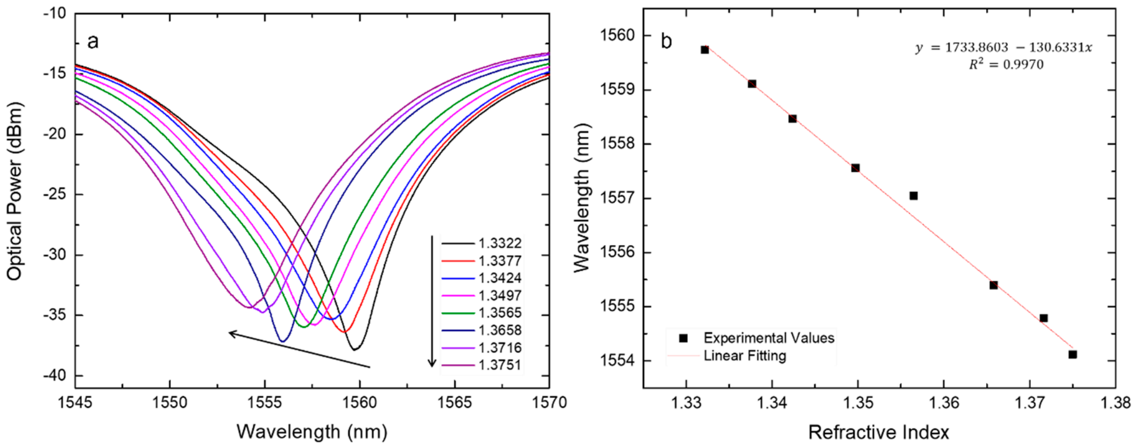

Supplementary figure S2 – (a) Attenuation band spectra for different ethylene glycol refractive index solutions; and (b) respective calibration curve plotting the wavelength position of each attenuation band (n = 10) as a function of the refractive index.

Supplementary equation S1, where **FOM** is the Figure of Merit, **S** is the sensitivity of the sensor, and **FWHM** is Full Width at Half Maximum of the resonance wavelength band.

$$FOM = \frac{S}{FWHM} \qquad \text{(Equation S1)}$$

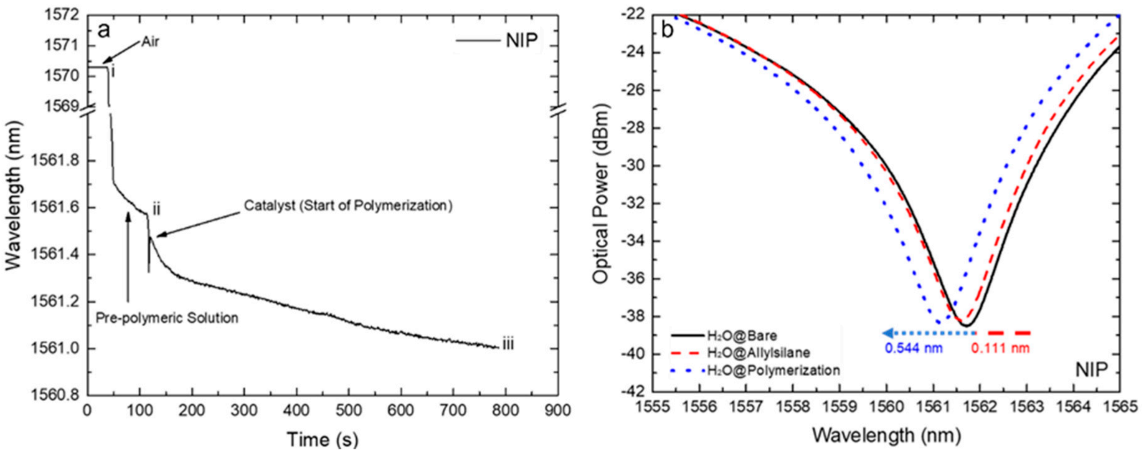

Supplementary figure S3 - (a) Real timeline of the resonance wavelength position of the NIP polymerization process: i. the pre-polymeric solution was placed; ii. the catalyst was placed; iii. the polymerization process was stopped. (b) Acquired spectra showing the wavelength resonance shift after allyl-silanization process (dashed line) and MIP layer formation (dotted line). Solid line is the bare fiber spectrum in water.

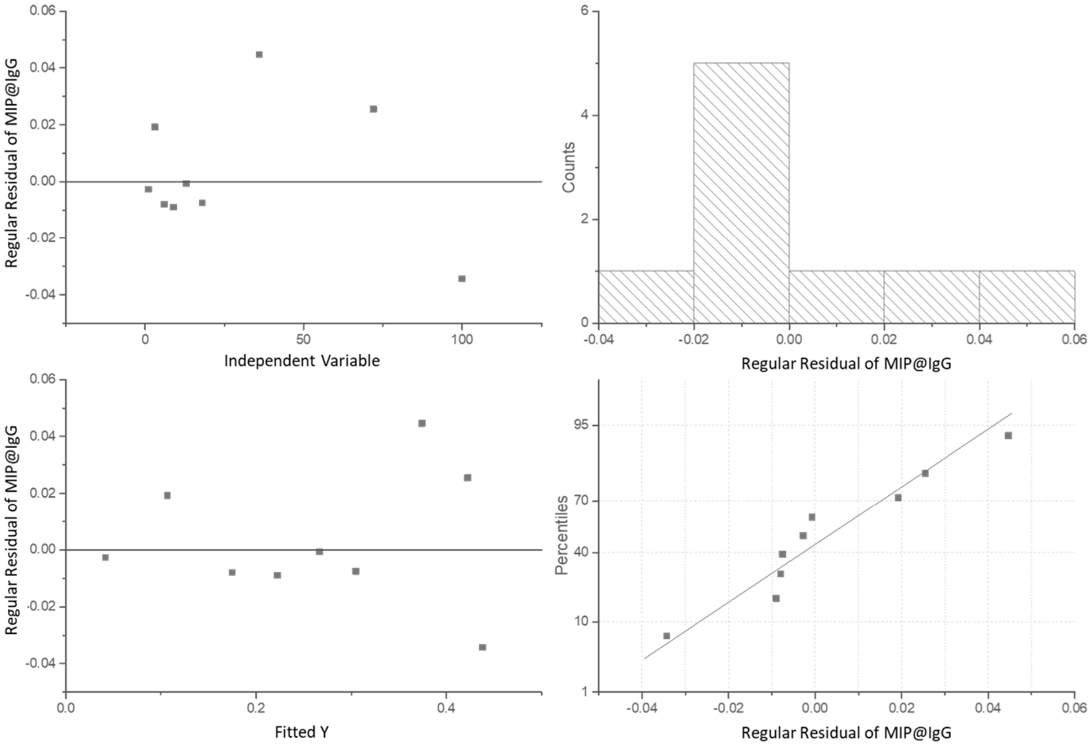

Supplementary figure S4 – Residual Plots resulted from the Hill fitting of the MIP@IgG curve.

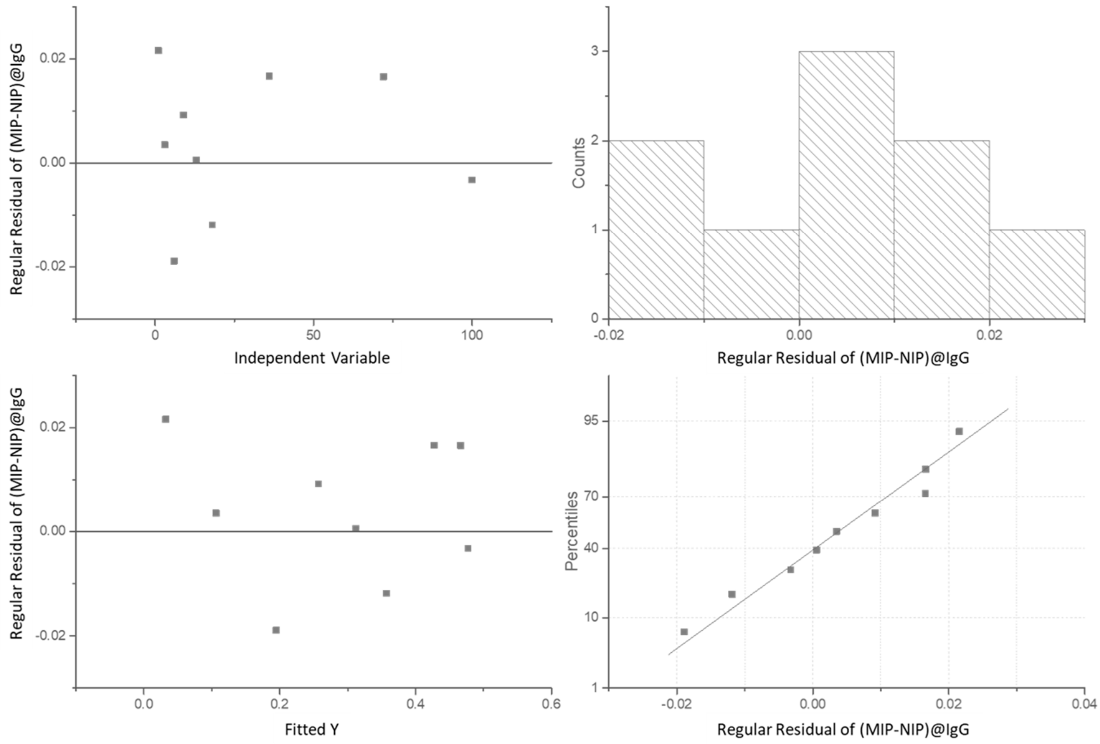

Supplementary figure S5 – Residual Plots resulted from the Hill fitting of the (MIP-NIP)@IgG curve.

Supplementary equation S2, Hill model equation where $\Delta\lambda_c$ is the wavelength variation at the concentration C; $\Delta\lambda_{max}$ is the wavelength variation at the saturation point (or response to the infinite); K is the ligand concentration at the $1/2$ $\Delta\lambda_{max}$; and n is the Hill coefficient.

$$\Delta\lambda_c = \frac{\Delta\lambda_{max} \times C^n}{(K^n + C^n)} \qquad \text{(Equation S2)}$$

Supplementary equation S3, where β_2 relates with the selectivity and activity of the ions, β_1 is the slope parameter, and σ is the standard deviation of the blank.

$$LOD_{S/N} = \beta_2 \left(10^{\frac{3\sigma}{\beta_1}} - 1\right) \qquad \text{(Equation S3)}$$

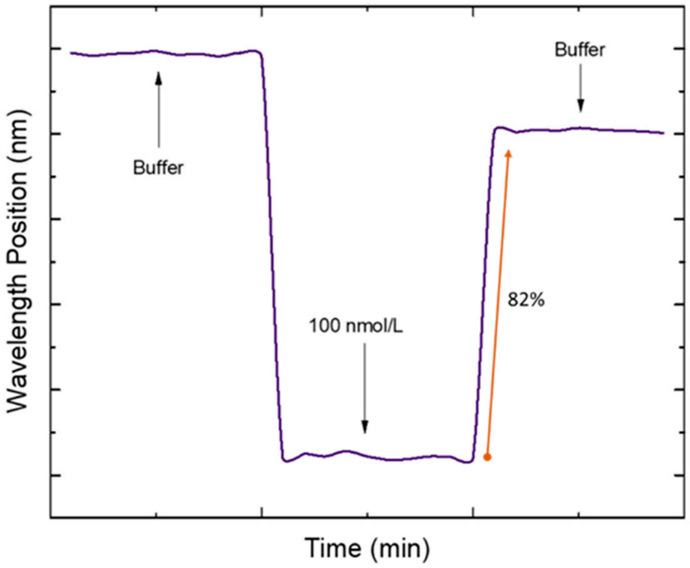

Supplementary figure S6 – Wavelength peak position of the differential (MIP-NIP) shifting over time throughout reversibility trials.
